# Label-free *in vivo* Raman microspectroscopic imaging of the macromolecular architecture of oocytes

**DOI:** 10.1038/s41598-017-08973-0

**Published:** 2017-08-21

**Authors:** Philip Heraud, Katarzyna Maria Marzec, Qing‒Hua Zhang, Wai Shan Yuen, John Carroll, Bayden R. Wood

**Affiliations:** 1 Centre for Biospectroscopy, School of Chemistry, Monash University, Clayton, VIC, 3800 Australia; 2 Department of Microbiology, Monash University, Clayton, VIC, 3800 Australia; 3 Biomedicine Discovery Institute, Monash University, Clayton, 3800 VIC, Australia; 4 Jagiellonian Centre for Experimental Therapeutics (JCET), Jagiellonian University, Bobrzynskiego 14, 30–348 Krakow, Poland; 5 Centre for Medical Genomics-OMICRON, Jagiellonian University Medical College, Kopernika 7C, Krakow, 31–034 Poland

## Abstract

Confocal Raman spectroscopy (CRS) can provide information about oocyte competency through measurement of changes in the macromolecular architecture during oocyte development and maturation. Hitherto most spectroscopic studies have been limited to fixed oocytes due to the inherent difficulties working with live cells. Here we report the first three-dimensional images of living murine oocytes using CRS. We show that fixation induces significant changes in the macromolecular chemistry compared to living oocytes. A band at 1602 cm^−1^, assigned to a marker for mitochondria function was found in living oocytes but absent from fixed oocytes providing an *in vivo* marker. Fixation resulted in significant changes in protein and nucleic acid bands and the spatial distribution of organelles. Raman imaging of Metaphase I and II (MI, MII) and germinal vesicle stage oocytes showed changes in nuclear organisation and cytoplasm macromolecular architecture during these development and maturation stages related to changes in chromosome condensation, mitochondria aggregation and lipid droplet numbers.

## Introduction

The mammalian oocyte undergoes a sequence of nuclear and cytoplasmic changes during the process of oocyte maturation that are essential for fertilization and subsequent development^1^. The changes involve a highly ordered progression through the meiotic cell cycle from prophase of meiosis I to metaphase of meiosis II (MII). This progression is accompanied by major changes in nuclear architecture as chromatin condenses and the nuclear membrane breaks down. The formation of the first meiotic spindle then takes about 5 hours before it migrates to the cortex and proceeds to separate the chromosomes equally between the oocyte and the first polar body. The maternal chromosomes then reform a second meiotic spindle and the oocyte remains arrested until fertilization triggers the completion of MII and entry into embryogenesis. This progression through meiosis is accompanied by major changes in cellular architecture that extends beyond the cell cycle machinery. The endoplasmic reticulum and mitochondria aggregate around the developing meiotic spindle and then disperse through the oocyte after it reaches metaphase II. The cellular biochemistry of the oocyte also undergoes significant changes because kinase and phosphatase activities drive the cell cycle progression, which is accompanied by changes in oocyte metabolism, energy consumption, redox state and ionic regulation. This knowledge points to a highly dynamic process, the precise details of which remain largely unknown. A deeper understanding of the architectural and biochemical changes will provide an improved understanding of developmental mechanisms as well as being of practical of importance to improving the outcomes of assisted reproductive techniques where better understanding of these processes may allow more efficient identification and selection of developmentally-competent oocytes.

A significant bottleneck in this area is that there are no current methods available for probing the phenotypic changes occurring during oocyte development and maturation. Clinically, this is largely limited to observations of morphological change. Analysis of transcriptome pools has been explored in recent years showing variability in the phenotypes of human oocytes drawn from the same patient^1^. A number of approaches including spectroscopic analysis of culture media and metabolomics have been proposed as reliable indicators of oocyte and embryo viability, and although finding crude indicators of embryo viability^2^, have not yet provided sufficiently reliable predictive tests of oocyte competence.

Recently new molecular imaging approaches based on vibrational spectroscopy have emerged that provide subtle compositional information across a broad range of macromolecules obtained directly through spectroscopy without requiring any contrast agents, and enable simultaneous image reconstruction of multiple components. In this suite of spectroscopic imaging approaches, Raman confocal microspectroscopy is particularly powerful allowing the possibility of *in vivo* charaterisation of oocyte molecular composition at sub-micron spatial resolution in three dimensions.

There are only a few studies employing Raman spectroscopy to study oocytes including: using the technique to study changes across different maturation stages and after *in vitro* maturation^3, 4^ and examining the effects of oxidative damage and oocyte age^5^. None of these works employed the 3D optical sectioning capability of the technique^6^ and therefore could not assess the cellular molecular architecture of oocytes in an unambiguous manner. More importantly, all previous studies examined fixed oocytes with the possible perturbing effect of fixation on cell chemistry and structure not determined except in the work by Davidson *et al*.^2^, who attempted to address this issue comparing two dimensional maps of fixed and living oocytes, but reported little or no changes caused by fixation. In contrast, here we show marked differences between fixed and live oocytes concluding the previous study was limited by measurements with restricted spectral range and sensitivity.

Here we combine Raman three dimensional imaging using optical sectioning and multivariate analysis to investigate fixed and living oocytes in all developmental stages, and assigned an important *in vivo* spectroscopic maker band. The potential of *in vivo* confocal Raman microspectroscopy to assess oocyte competency in the context of assisted reproductive therapies is discussed.

## Results

### Imaging of live versus fixed oocytes

**Figure 1:**
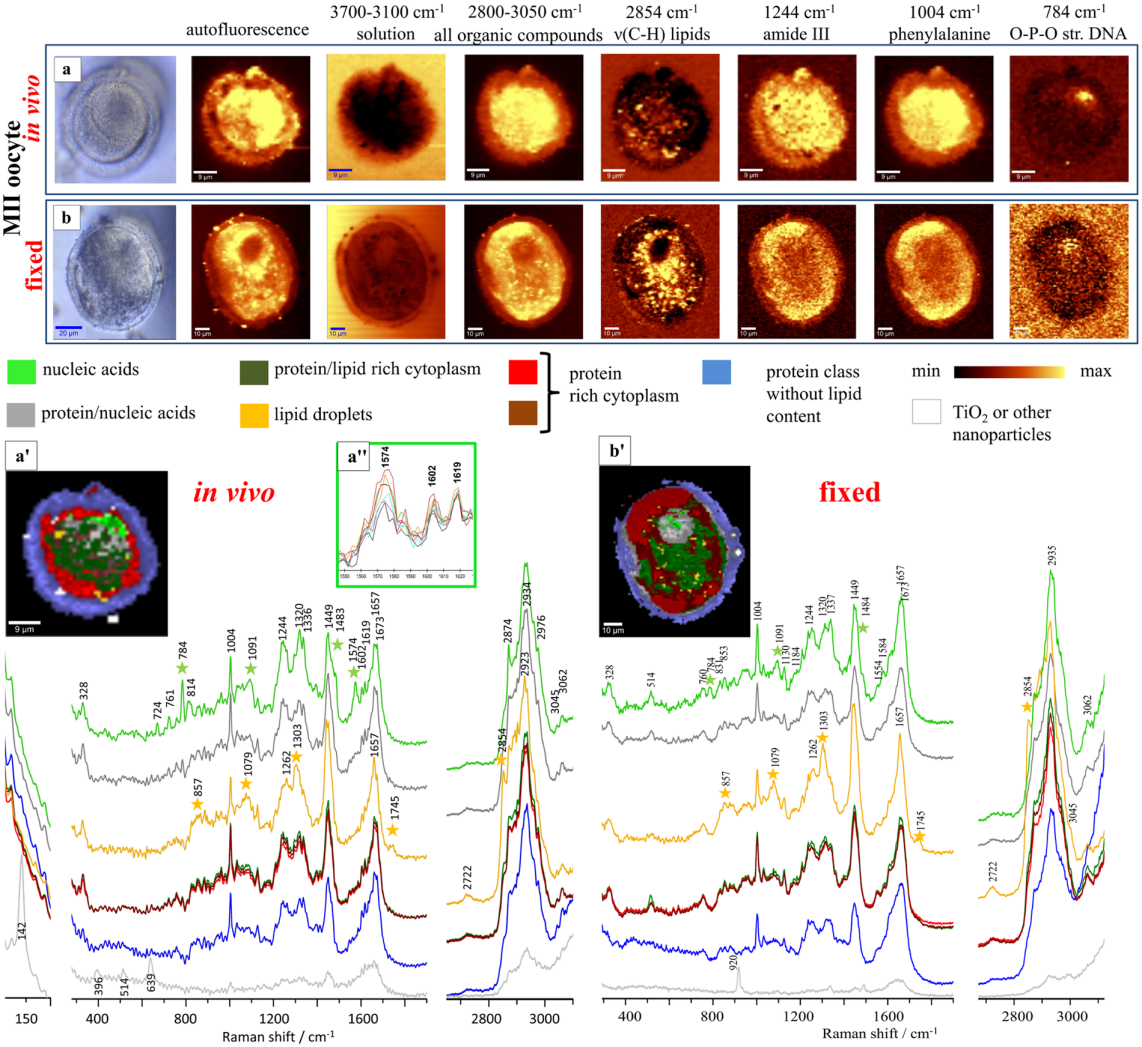
A photomicrograph of (**a**) functional and (**b**) fixed oocytes investigated with the use of air objective (100×/0.90NA) in the MII stages; Integration Raman maps of a specific bands were obtained with 532 nm laser wavelength and with a sampling density of 1 μm (maximal spatial resolution equal to 0.33 μm); K–means Clustering (KMC) results with the 8 main classes were presented with average spectrum for each class. In a” we have additionally presented the zoom-in of the spectral region showing the band at 1602 cm^−1^ for the single spectra extracted from the nucleic acids class, which is  onl﻿y observed in the *in vivo* state﻿. The Raman intensities in the region of 300–1900 cm^−1^ were scaled by factor of 2 comparing to CH-stretching region and lower region below 300 cm^−1^. A spectral class corresponding to substrate signal observed surrounding the oocytes was removed from the image (black pixels).

Raman confocal images of 8 MI, 8 MII and 4 GV murine oocytes were acquired at 1 μm intervals in the x,y plane. K-means clustering was performed to a level of 7 classes. The mean extracted Raman spectra for each of the 7 classes are shown and coded with the same colours in the images (Fig. 1).

We found that the K-means clusters cluster could be correlated to the known structure of oocytes (Fig. 1). Mean extracted spectra from each cluster were compared to investigate the macromolecular composition. Although, the average spectra from different classes do not represent pure component spectra, they can be interrogated visually to define the biological origin of the cluster region in question. The cluster colour-coded light green in Fig. 1 was clearly related to chromosomal material in both fixed and live cells, as the mean extracted spectra had prominent bands that could be assigned to nucleic acids (Fig. 1 and Table 1). These included bands at 1574, 1483, 1336, 1262, 1212, 1091, 784, 760 and 723 cm^−1^ (Fig. 1 and Table 1). The size and position of the light green cluster correlated well with condensed metaphase chromosomes observed in MII oocytes^7^. In the live oocyte the chromosomal material was located in the polar region of the cell. The position of the chromosomal material was clearly defined by the Raman intensity maps based on the band at 784 cm^−1^ (Fig. 1).

**Table 1:** Observed wavenumber values (cm^−1^) for the most prominent bands of functional and fixed oocytes with assignments^8–12^ obtained with the 532 nm laser line.

Fixed	Functional	Band Assignment	Compound
Wavenumber/cm^−1^			
3062		aromatic residues ν(CH)	proteins
−	3045		
2934(5)		aromatic and aliphatic ν(CH)	proteins
2874		aliphatic ν(CH)	proteins
2854		ν_sym_ (CH_3_)	lipids
2722		aliphatic ν(CH_2_,CH_3_)	lipids/proteins
1745		ν(C=O) ester	lipids
1673 1655–1680		Amide I	proteins
1657		ν(C=C)	lipids
−	1619	ν(C=C), Tyr, Trp	proteins
−	1602	mitochondrial activity/ ν(C=C), Phe, Tyr	Ca^2+^ influence/proteins
1584	1574	G, A/Phe	nucleic acids/proteins
1554			
1483(4)		G, A/CH def	nucleic acids/proteins
1449		C-H_2_ def/ CH def	proteins/lipids
1336		A,G/ C-H def	nucleic acids/proteins
1320		G/ C-H def	nucleic acids/proteins
1303		Amide III/ CH_2_ twist	proteins/lipids
1262		T,A/ C-H bend, Amide III/=CH bend	nucleic acids/proteins/lipids
1244		Amide III	proteins
1212		T,A/ Amide III	nucleic acids/proteins
1184		Tyr, Phe, C-H bend	proteins
1157		ν(C-C,C-N)	proteins
1130		ν(C-N)	proteins
1091		ν_sym_(O-P-O)/ ν(C-N)	nucleic acids/proteins
1079		chain ν(C-C) *gauche*	lipids
1063		chain ν(C-C) *trans*	lipids
1004		sym. ring br. Phe	proteins
857		Tyr. ring br./ ν_sym_(C-C-N^+^)	proteins/lipids
	814	ν_asym_(O-P-O) / ring br. Tyr	RNA /proteins
784		ν(O-P-O)/U, T, C	DNA/nucleic acids
760		T/ ring br. Trp	nucleic acids/proteins
723		A	nucleic acids
514		S-S brigs	proteins
639		TiO_2_	anatase nanoparticles
514		TiO_2_	anatase nanoparticles
396		TiO_2_	anatase nanoparticles
142		TiO_2_	anatase nanoparticles

^a^ν – *stretching*, def – *deformation*, br – *breathing*, sym – *symmetric*, asym – *asymmetric*, Phe – phenylalanine; Trp – *tryptophan*; Tyr- tyrosine; T – thymine; A – adenine; G – Guanine; C – cytosine; U – uracil.

Contiguous to the light green coded cluster assigned to condensed chromosomes in both live and fixed cells, was a grey cluster where the mean extracted spectra showed intense protein and nucleic acid bands. These included the amide I and amide III modes at 1657 and 1244 cm^−1^ (Table 1). A larger cluster (colour coded dark green) was contiguous to the light green and grey clusters. The average spectra for this cluster showed that it was deficient in nucleic acids compared to the light green and grey cluster regions, with the mean extracted spectra indicating a mixture of proteins and lipids. The lipid bands appear at 1745 and 2854 cm^−1^ and the protein bands at 1657 and 1244 cm^−1^ (Fig. 1 and Table 1). The mixture of protein and lipid components as well as its position adjacent to the nuclear material was good evidence to suggest that the dark green cluster corresponded to endoplasmic reticulum that has been shown to surround the first meiotic spindle that carries the chromosomes during cell division^13^.

The mean extracted spectra from the largest clusters in both fixed and live oocytes (colour coded red and brown) surrounding the light and grey clusters were dominated by protein bands (Amide I and III and phenylalanine bands, Fig. 1 and Table 1) whereas bands attributed to nucleic acids and lipids were weak or absent. These clusters correspond to protein rich cytoplasmic regions not containing golgi systems or extensive endoplasmic reticulum observed in MII oocytes^13, 14^.

Pronounced differences were observed in the spectral profile of bands associated with the nucleic acid and protein rich clusters described above. The most distinctive differences were observed in the region connected with backbone geometry of nucleic acids and phosphate ion structure (800–1200 cm^−1^), nucleotides (1200–1600 cm^−1^) as well as changes in protein features (Fig. 1, Table 1). The bands connected with stretching vibrations of C-C and C-H modes of lipids and proteins were found to be unchanged between fixed and live MII oocytes. Similarly, protein amide vibrations, such as the amide I band and amide III band did not significantly change due to fixation. However, a visible impact of fixation on proteins was manifest in band at 514 cm^−1^, which was assigned to the vibrations of S-S bridges (Fig. 1, Table 1). This band was most prominent in fixed oocytes but entirely absent in live cells. By contrast, a band prominent in living MII oocytes but entirely absent in all fixed ones was observed at 1602 cm^−1^ (Fig. 1, Table 1), and assigned to functional mitochondria^15, 16^.

Bands which can be assigned to the ring breathing modes of the DNA bases (thymine, cytosine), as well as PO_2_^−^ stretching vibrations of the DNA backbone could be observed at 784 cm^−1^ from DNA in both functional and fixed oocytes. However, in spectra of fixed MII, its intensity is significantly reduced. This was also confirmed by the similarly reduced intensity of the band at 1091 cm^−1^, which is a mode of the symmetric PO_2_^−^ stretching vibration of the DNA backbone. Raman bands observed in live oocytes at 1483, 1336 and 1320 cm^−1^ connected with nucleic acids vibrations were also stronger in intensity compared to fixed cells (see Table 1, Fig. 1). Besides variations in band intensity between fixed and live oocytes, shifts and appearance of new bands connected with nucleic acids were also observed. For example, the strong band assigned to vibrations of guanine and adenine, observed at 1574 cm^−1^ in living oocytes was changed to a weaker doublet at 1554 and 1584 cm^−1^ in fixed cells. The band assigned to the asymmetric PO_2_^−^ stretching vibration of the RNA backbone, which was observed at 814 cm^−1^ in live oocytes was absent in fixed cells.

**Figure 2:**
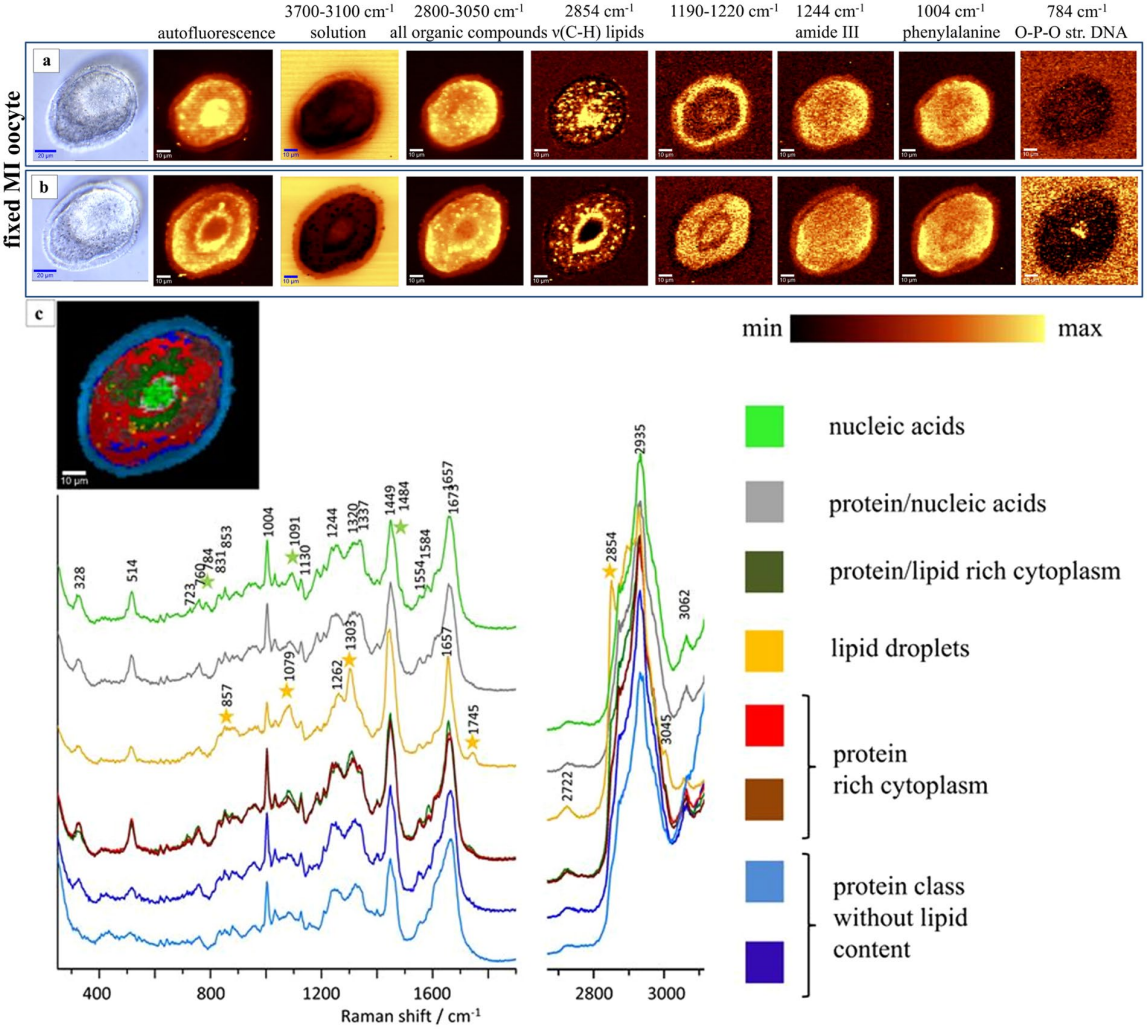
**A** showing micrographs through two horizontal image planes separated by 10 µm through a fixed MI oocyte. (a) Corresponds to the plane 10 µm above a central plane through the nuclear region shown in (b). The heat maps in (a and b) compare autofluorescence and maps showing the intensity of Raman spectral bands related to oocyte composition. The yellow color corresponds to the highest relative intensity of integrated band related to the distribution of different compounds or group of compounds. Sampling densities were equal to 1 μm. (c) Shows K–means Clustering (KMC) results for the middle stack “b” with the 8 main classes were presented with average spectrum for each class. The Raman intensities in the region of 300–1900 cm^−1^ were scaled by factor of 2 comparing to CH-stretching region. A spectral class corresponding to substrate signal observed surrounding the oocytes was removed from the image (black pixels).

A lipid rich cluster colour coded yellow in Fig. 1 was identified. The localisation of spectra from this cluster to small regions typically equal to or less than 2 μm in width as well as the intense lipid bands associated with it (2854, 1740 cm^−1^) (Fig. 1, Table 1) indicated that these structures were most probably lipid droplets that are known inclusions in MII oocytes^17, 18^. By contrast with nucleic acid bands, the most important marker bands of lipids observed at 2854, 1745 and 1303 cm^−1^ (Fig. 1, Table 1), showed little or no differences based on fixation. However, there was a difference in the spatial distribution of the lipid droplets within live and fixed MII oocytes. This was revealed in the cluster maps (Fig. 1) and by imaging the ν_sym_(CH_3_) stretching mode from lipid moieties at 2854 cm^−1^. In fixed oocytes the lipid rich bodies were clustered around nucleic acid rich regions identified as condensed chromosomes. By contrast lipid droplets in live oocytes were randomly distributed throughout the cytoplasm (Figs 1 and 2). There were no lipid bodies found in the same region where the nucleic acids were concentrated. Indeed, nucleic acid rich regions where the 784 cm^−1^ band from nucleic acids was most intense, showed a very weak 2854 cm^−1^ band indicating low lipid levels.

The final two clusters of biological origin colour-coded dark blue and purple in Fig. 1 are located at the periphery of both living and fixed MII oocytes. The mean extracted spectra for these two clusters showed intense protein bands, with no evidence of lipid or nucleic acid bands. The position, size (~5 μm in width) and the protein rich-lipid poor nature of this region corresponded to oocyte zona pellucida. The zona pellucida was clearly identified using light microscopy and corresponds exactly to the dark blue and purple clusters.

A white cluster with a distinct bands at 142, 396, 514 and 639 cm^−1^ of non-biological origin were assigned to titanium dioxide particles (Fig. 1a; Table 1) with the characteristic Raman spectrum for the anatase (tetragonal structures of TiO_2_)^19^. The nanoparticles observed in the zona pellucida of the fixed MII oocyte are presented in the Fig. 1b and show a characteristic sharp band at 920 cm^−1^, tentatively assigned to acetate particles^20^. Such nanoparticles were previously observed in different Raman studies of biological samples and are recognized as common contamination coming from paints, coatings, plastics and other commonly used products^19^.

Figure 2 presents the Raman integration maps as well as cluster analysis for the fixed MI oocyte. Unfortunately, we were not able to obtain Raman maps for the living MI stage with the chromosomal region visible, as the chromosomes are condensed and it is difficult to locate the exact plane that targets the nucleus in this stage. Figure 2 shows the average Raman spectra for the K-means clusters of fixed MI oocyte. These show very similar information, to the fixed MII oocyte imaging shown in Fig. 1, including the absence of the band at 1602 cm^−1^, the S-S bridging mode from proteins at 514 cm^−1^ and changes to nucleic acids bands, indicating that these spectroscopic changes are a generalised effect of fixation.

### Imaging of live oocytes from different developmental stages

**Figure 3:**
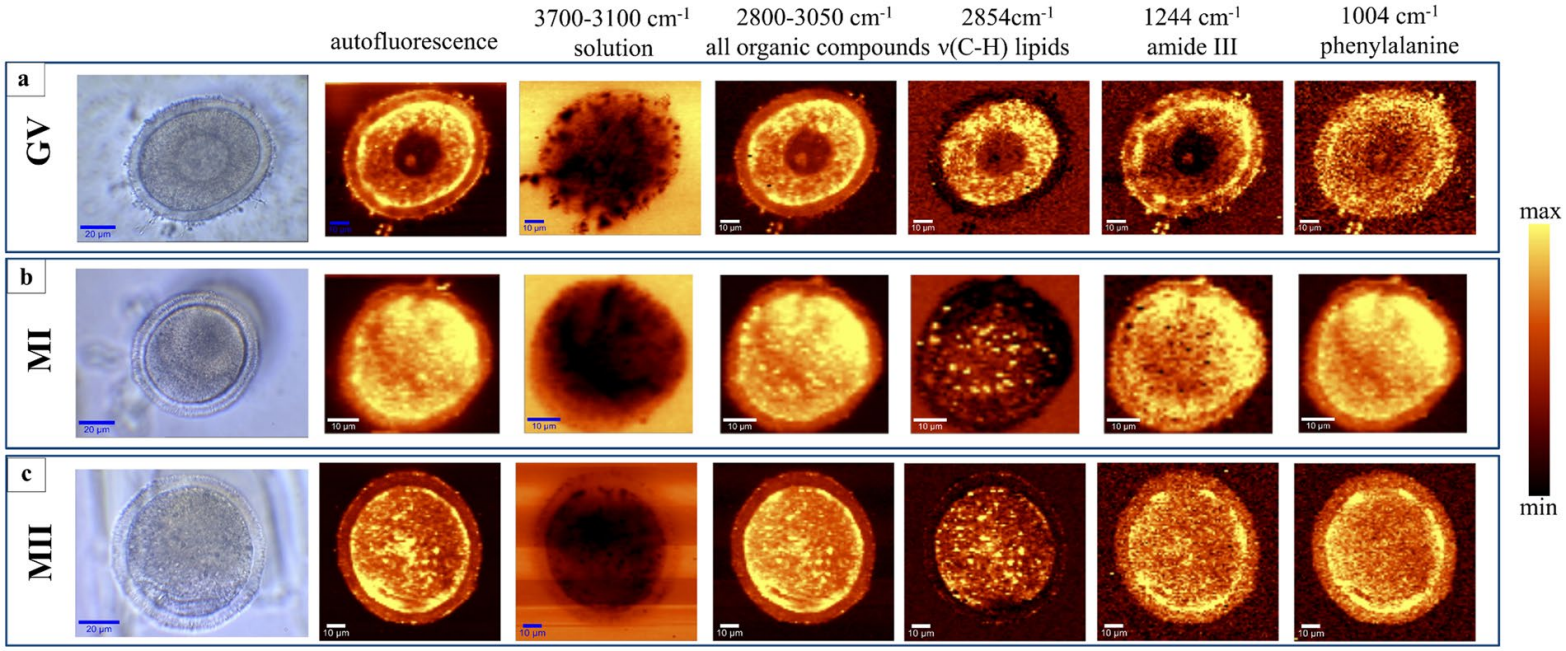
Showing micrographs through the central plane of live GV (**a**), MI (**b**) and MII (**c**) oocytes compared with maps of autofluorescence and maps showing the intensity of Raman spectral bands related to oocyte composition. The yellow color corresponds to the highest relative intensity of autofluorescence or integrated band related to the distribution of different compounds or group of compounds. Spectra used to create the maps were acquired at 1 μm intervals.

Living oocytes from GV, MI and MII stages were compared using Raman microspectroscopy in Fig. 3. Images based on Raman band intensity showed live GV oocytes were noticeably distinct compared to live MI and MII oocytes. The intensity of the C-H stretching bands (2800–3050 cm^−1^; Table 1) are attributed to organic matter in GV oocytes, which show a circular central region approximately 30 μm in diameter where the bands were much less intense. This region correlated to the GV prophase nucleus. A small circular region approximately 5 μm in diameter showed intense C-H stretching bands and higher protein amide III band intensity (1244 cm^−1^; Fig. 3) compared to the rest of the putative nuclear region, and was identified as the nucleolus. Chemical maps based on band intensity for live MI and MII oocytes were similar showing a fairly homogeneous distribution of lipid rich bodies identified as lipid droplets in the live MII oocyte shown previously in Fig. 1. Consistent with the identification as lipid droplets these lipid rich regions had a very low intensity amide III protein band (Fig. 3), corroborating the assignment to lipid droplets.

### Three dimensional imaging of living oocytes

**Figure 4:**
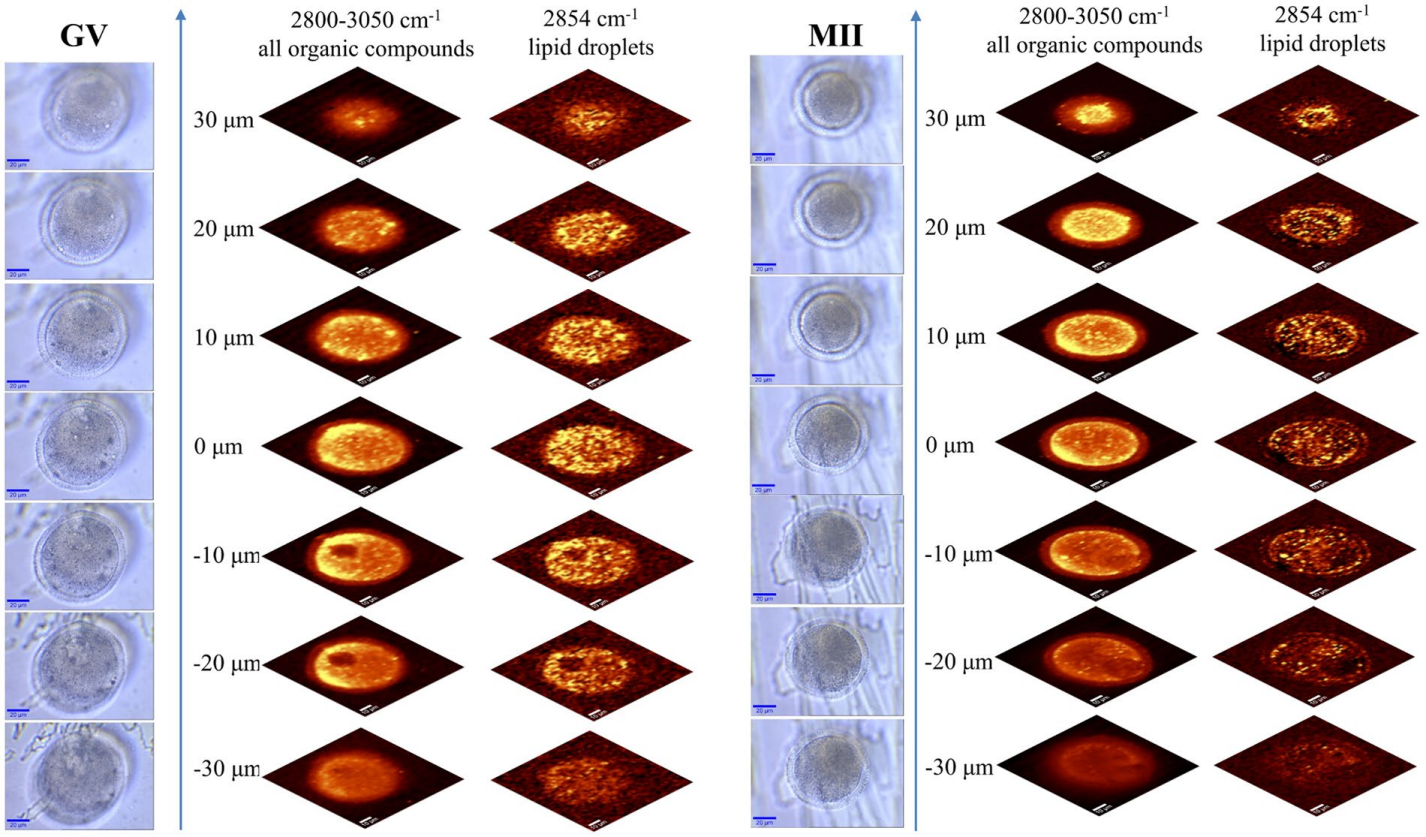
Confocal “optical slicing” through living GV (left side) and MII (right side) oocytes. The images acquired at 7 different vertical positions through the cells, 10 μm apart in each case, are from left to right for each oocyte: visible images; integration Raman maps of bands intensity related to organic compounds; and lipids, respectively. The yellow color corresponds to the highest relative intensity of the integrated band related to the distribution of compound or group of compounds. Spectra were acquired at intervals of 1.5 μm in each two dimensional map.

The composition of cells including oocytes varies throughout the cell volume. Accordingly, we employed the confocal ability of the Raman microscope to acquire spectroscopic information through different planes through living GV and MII oocytes. This has been proposed before with FTIR images of tissue sections^21^. Figure 4 shows maps based on band intensity of the C-H stretching band region (2800–3050 cm^−1^) corresponding to the concentration of organic matter, and maps of the intensity of the lipid band at 2854 cm^−1^. These were done at 10 μm intervals in the z-direction, through the central axis of the cells and through positions up to 30 μm above and below the central plane (Fig. 4). The composition of the oocyte changes considerably with sampling height throughout the cell. This is particularly evident in the GV cell with the nucleus identified in the same manner as in Fig. 3 as a region of lower organic concentration observed only in two planes 10 and 20 μm below the central plane of the cell. Lipid droplets, identified by the intensity of the band at 2854 cm^−1^ were homogenously distributed in all planes of both the GV and MII oocytes. There was no evidence of lipid inclusions within the nucleus in the GV nucleus.

## Discussion

### New possibilities provided by live macromolecular imaging

Oocyte maturation and development and subsequent achievement of competency is accompanied by a characteristic sequence of phenotypic changes. Raman confocal microimaging allowed high resolution imaging of cellular architecture across multiple molecular components providing a comprehensive assessment of the oocyte compositional phenotype. This was clearly evident by imaging organelles and structures identifiable as typical for GV, MI and MII oocytes. These included non-condensed and condensed DNA, mitochondria, cytoplasm, zona pellucida. Importantly, the macromolecular composition in the live condition could be probed directly at the micron scale. Comparing fixed and live oocytes demonstrated that in many cases the morphology of cellular structures appeared little altered, e.g. the GV nucleus, however the macromolecular chemistry was altered drastically.

### Raman imaging reveals structural and compositional differences between live cells and fixed cells

The only study to date using vibrational spectroscopy to probe the molecular architecture of oocytes^4^ was been limited in two fundamental ways. First it focused primarily on fixed oocytes. Second it was limited to imaging of cells in two dimensions. This is the first study that provides a comprehensive analysis of live oocytes in three dimensions.

The comparison of living and fixed cells presented here demonstrates unequivocally that aldehyde fixation changed both the structure of oocytes and the molecular composition. Compositional change was most evident in changes to nucleic acid and to protein structure. The strong diversity of spectral characterization related to nucleic acid components between functional and fixed MI oocytes indicated degradation of the oocyte DNA composition due to fixation. These observations are is in line with previous studies, which suggested that the nucleic acids may be degraded during fixation or made resistant to extraction because of cross-linking of histones^22^. Evidence of changes to nucleic acids is supported by reports that formaldehyde and glutaraldehyde may interact with all cellular nucleic acids^23^. We also observed changes to proteins in fixed cells compared to live cells, corroborating previous studies using Raman spectroscopy that aldehyde fixation causes changes related to proteins^24^. We observed changes to protein composition related to the formation of additional disulfide bridges under formalin fixation. The additional protein disulfide bridges created due to fixation may be connected with the oxidation of thiol groups of amino acids in such conditions, mainly by oxidizing cysteins, however this needs to be confirmed with further studies.

By contrast with its effects on nucleic acids and proteins, we observed very little changes in the chemical composition of lipids due to fixation. These changes are supported by other studies that have been previously reported that aldehyde fixatives appear not have an impact on carbohydrates or lipids^25^. However, we did find that fixation did cause changes in lipid distribution in oocytes. This study showed lipids to be concentrated mainly as lipid droplets that were distributed evenly throughout the cellular volume in all living oocytes that were studied, apart from their absence within the nucleus in GV cells, what is in agreement with previous studies^26^. By contrast, lipid droplets appeared consistently to be condensed surrounding the nuclear material in fixed cells. The reason for the change in lipid distribution caused by fixation is not understood and needs further investigation. This comparison clearly demonstrates the importance of observing cells in there *in vivo* state when drawing conclusions about their structure as related to function. Wood *et al*.^4^ showed centralised congregation of lipid droplets in meiotic oocytes, which we can now assume was related to fixation as opposed to any functional significance.

Changes observed in this study attributed to aldehyde fixation is in direct contrast to a previous study^3^. We can see two major reasons for why the changes observed here were not seen in the previous study leading to the authors concluding that the information from formalin-fixed oocytes was identical to living oocytes. First, our study employed cluster mapping to identify regions of spectral similarity in the oocyte. Average spectra from these clusters derived from hundreds of spectra will have a much higher signal-to-noise ratio that individual spectra that were compared in the previous study allowing small but important spectral differences, such as changes in nucleic acid signatures to be observed. Secondly, our study included a much broader spectral range compared to the previous study enabling the identification of a band at 514 cm^−1^ in fixed oocytes assigned to the S-S bridging mode.

The viability of all our unfixed oocytes in this study was confirmed by the presence of an *in vivo* marker band at 1602 cm^−1^, previously reported in living HeLa cells, peripheral blood lymphocytes, human mesenchymal stem cells and bovine chondrocytes^16^. This is the first report of this band enabling discrimination of living oocytes from fixed, non-living oocytes. This band, previously described as a “signature of life”^15, 27^, is still to be assigned unequivocally, but appears to be related to a mitochondrial component that is connected with Ca^2+^ uptake. The “band of life” reported for a living yeast cells^27^ had a FWHM comparable to the band at 1655 cm^−1^ as well as being much stronger compared to band appearing at the same position in living oocytes. As presented in the Fig. 1a”, the extracted single spectra from the nucleic acids class highlights the “band of life” at 1602 cm^−1^ only observed in living oocytes. This band typically is less intense and has a smaller FWHM compared to that observed in live yeast cells, for example. This band was not observed in the Raman spectra of any fixed oocytes we studied confirming the view that it is a marker of living oocytes. As changes in mitochondrial number and activity are known to accompany key changes in oocyte development, it may be possible to use the intensity of this band as an indicator of these key events.

### Three dimensional compositional imaging of live oocytes and its diagnostic potential

Visualization of the three dimensional architecture of cells usually requires physical sectioning of the cell, imaging of the two dimensional images and reconstruction of the two dimensional data to form a three dimensional representation. Confocal microscopy, on the other hand, allows information to be gathered from a very narrow and defined depth range in the cell whilst excluding information from other regions, referred to as “optical sectioning”^28^. When this is combined with Raman spectroscopy cellular molecular architecture information can be obtained in three dimensions whilst maintaining the cell in an intact *in vivo* condition. Furthermore, software can be employed to combine the two dimensional molecular images to form three-dimensional representations of the molecular architecture. This study shows that this approach can be achieved with live oocytes. Three dimensional imaging has the advantage of overcoming ambiguities that may arise, for example, when imaging is done in transmission in two dimensions. An example is the study by Wood *et al*. (2008)^4^ that observed small central lipid deposits superimposed on the nuclear region of GV oocytes using synchrotron FTIR microspectroscopy. The two dimensional nature of the imaging used rendered it impossible for the authors to conclude whether the lipid deposits were in the nucleus or simply in the cytoplasm overlying the nucleus. In this study using confocal Raman microscopy the nucleus of GV nuclei were readily identified in confocal two dimensional maps as regions of lower concentration of organic material, consistent with previous measurements showing low macromolecular concentration in GV nuclei in comparison with surrounding cytoplasm^4^. By mapping lipid distribution using the intensity of characteristic lipid bands it was clear that the nuclear region was completely devoid of lipid in successive two dimensional optical slices through the nucleus.

## Conclusions

We have demonstrated that confocal Raman microspectroscopy can provide high resolution imaging of macromolecular architecture of living oocytes defined in three dimensions. The measurements can be regarded as non-invasive because no contrast agent was applied and the laser power was low and only short acquisition times were required for the measurement. We have demonstrated unequivocally that aldehyde fixation causes marked changes in both macromolecular composition and distribution, drawing into question the conclusions about oocyte macromolecular composition based on Raman spectroscopy of fixed oocytes. Indeed, this work demonstrates clearly that efforts to identify signatures of oocyte competence via oocyte macromolecular composition by Raman spectroscopy must necessarily be done using *in vivo* measurements.

## Methods

All research was performed in accordance with national and university guidelines and with approval of the Monash University Animal Ethics Committee.

### Functional and fixed oocyte preparation

For *in vitro* maturation, GV oocytes were collected in M2 (Sigma) containing 200 µM 3-isobutyly- 1-methylx- anthine (IBMX). Oocyte maturation was performed in drops of M16 medium (Sigma) under mineral oil (Sigma) at 37 °C in a humidified atmosphere of 5% CO_2_ in air. GV oocytes were allowed to mature for 8 h in IBMX-free M16 media in order to obtain MI samples. For collection of MII stage oocytes, mice were superovulated by sequential intraperitoneal injections of 10IU pregnant mare’s serum gonadotropin (PMSG, Intervet) and 10IU human chorionic gonadotropin (hCG, Intervet) at timed intervals before oocyte collection. Oocytes of any stage were fixed using 4% paraformaldehyde made in phosphate-buffered saline (PBS) for 30 min at room temperature. All oocytes, live or fixed were washed into 0.9% saline for spectroscopy.

### Raman Confocal Microspectroscopy

Raman imaging of live and fixed murine oocytes was performed on a WITec confocal CRM alpha 300 Raman microscope. The spectrometer was equipped with an air–cooled solid state laser operating at 532 nm and a CCD detector, which was cooled to −65 °C. The laser was coupled to a microscope via a single mode optical fiber with a diameter of 50 um. The scattered radiation was focused onto a multi–mode fiber (50 um diameter) and a monochromator. The integration time for a single spectrum was 2 s with a spectral resolution of 3 cm^–1^. The monochromator of the spectrometer was calibrated using the Raman scattering line produced by a silicon plate (520.7 cm^−1^). For Raman map collection water immersive Nikon (x60/1NA) objectives was used. The power for all measurements of the laser at the sample position was ≤10 mW. The average 2D image of the oocyte was measured at ~80 × 80 µm^2^ with a step of 1 µm and the integration time for each collected spectrum equal to 2 s. In total it took 3.5 h to generate the 2D images. The 2D images presented in Fig. 4 were collected with the step of 2 µm that decreases the measurement time for one optical section to around 2 h.

### Data Analysis and Image Reconstruction

Raman data analysis was performed with Opus^TM^ and WITec software^TM^. Raman maps were generated based on the integration of marker bands and were obtained without pre–processing. Cluster analysis was carried out after cosmic ray spike removal, background subtraction (2^nd^ order polynomial in the spectral region of 100‒3500 cm^−1^). K–Means Clustering (KMC) results were obtained with the Manhattan distance algorithm and are complementary to the analysis based on the integration of the specific marker bands.
